# Causal relationship between gut microbiota and laryngeal cancer: a mendelian randomization analysis

**DOI:** 10.1016/j.bjorl.2025.101634

**Published:** 2025-04-30

**Authors:** Kaiyan Yi, Yu Huang, Yun Jiang, Lingling Zhou

**Affiliations:** Department of Otolaryngology-Head and Neck Surgery, Huadong Hospital, Fudan University, Shanghai, China

**Keywords:** Gut microbiota, Laryngeal cancer, The causal association, GWAS, Mendelian randomization

## Abstract

**Objective:**

Laryngeal cancer incidence is rising globally; the role of gut microbiota remains underexplored. This study aimed to establish a causal link between gut microbiota and laryngeal cancer to inform preventive and therapeutic strategies.

**Methods:**

Gut microbiota data from GWAS conducted by the MiBioGen consortium served as the exposure variable, with laryngeal cancer as the outcome variable. the exposure variable and the outcome variable were analyzed using Mendelian Randomization. The primary method was Inverse Variance Weighted analysis, with heterogeneity and pleiotropy assessed through Cochran's *Q* test, MR-Egger regression, and MR-PRESSO.

**Results:**

In the study, we identified five bacterial taxa with potential causal relationships with laryngeal cancer risk: Higher levels of Clostridiaceae1 (OR = 0.9993, 95% CI 0.9986–0.9999, *p* = 0.0463) and Turicibacter (OR = 0.9995, 95% CI 0.9989–0.9999, *p* = 0.0384) were linked to reduced cancer risk, while Mollicutes RF9 (OR = 1.0010, 95% CI 1.0003–1.0016, *p* = 0.0027), Euryarchaeota (OR = 1.0004, 95% CI 1.0001–1.0007, *p* = 0.0234), and Cyanobacteria (OR = 1.0005, 95% CI 1.0000–1.0009, *p* = 0.0464) were associated with increased risk.

**Conclusion:**

Our findings suggest a causal relationship between gut microbiota composition and laryngeal cancer risk. Clostridiaceae1 and Turicibacter may play a protective role, while Mollicutes RF9, Euryarchaeota, and Cyanobacteria could contribute to increased cancer susceptibility. These insights highlight potential microbiome-based strategies for early detection, prevention, and therapeutic intervention in laryngeal cancer.

**Level of evidence:**

Level 5.

## Highlights

• This study establishes a link between gut microbiota and laryngeal cancer risk.
• Clostridiaceae1 and Turicibacter can reduce the risk of laryngeal cancer.
• Mollicutes RF9, Euryarchaeota and Cyanobacteria can improve laryngeal cancer’s risk.
• Targeting gut microbiota can offer strategies for laryngeal cancer’s treatment.

## Introduction

Laryngeal cancer is a malignant neoplasm that arises from the epithelial or connective tissues of the larynx and represents a significant subtype of Head and Neck Squamous Cell Carcinoma (HNSCC) observed worldwide.^1^, ^2^, ^3^ Histopathologically, the vast majority of laryngeal cancer, accounting for approximately 95%, are classified as squamous cell carcinoma. Furthermore, over 95% of diagnosed laryngeal cancer cases specifically present as Laryngeal Squamous Cell Carcinoma (LSCC).^4^, ^5^, ^6^ According to the most recent global cancer statistics, it was projected that over 19,270 new cases of Laryngeal Squamous Cell Carcinoma (LSCC) would be diagnosed in 2022, with an estimated 3,980 deaths occurring in the United States alone.^1^, ^7^, ^8^ Despite continued advancements in the diagnosis and treatment of laryngeal cancer, there has been minimal improvement in overall survival rates. This stagnation is largely attributed to the tumor’s aggressive characteristics, including its unpredictable incidence, high recurrence rates, and the trend toward younger age at disease onset, which imposes a substantial burden on individuals, families, and society at large.^9^ Unhealthy lifestyle practices, including tobacco use and alcohol consumption, along with infection by Human Papillomavirus (HPV), are the predominant risk factors driving the incidence of laryngeal cancer.^10^, ^11^ However, further research is necessary to clarify whether the gut microbiota is associated with the onset and progression of laryngeal cancer, as well as to identify the specific microbial communities involved in its development.

The human microbiome, which is located in distinct regions of the body, plays a critical role in various physiological processes. It is integral to the absorption of nutrients, maintenance of epithelial barrier function, detoxification of harmful substances, regulation of inflammatory and immune responses, and protection against pathogenic microorganisms.^12^, ^13^ Advancements in Next-Generation Sequencing (NGS) technologies have substantially enhanced our understanding of the human microbiome, especially the gut microbiome, which is predominantly composed of Bacteroidetes and Firmicutes.^14^ A healthy gut microbiota generates Short-Chain Fatty Acids (SCFAs) through the fermentation of dietary fibers, which play a pivotal role in maintaining gut acidity, fostering the growth of beneficial bacteria, preventing pathogen colonization, and promoting epithelial regeneration. Consequently, a balanced gut microbiome is crucial in mitigating chronic inflammation, obesity, metabolic syndrome, and diseases associated with cancer.^15^

The gut microbiota typically maintains a symbiotic equilibrium within the host, contributing to overall health and homeostasis. However, various factors can disrupt this microbial balance, including the use of medications, obesity, dietary habits, physical activity levels, and genetic predispositions.^15^ Intestinal dysbiosis, defined by a reduction in the diversity and stability of the gut microbiota, can result in the proliferation of pathogenic bacteria and the generation of detrimental metabolites. This imbalance disrupts normal immunological and metabolic functions and has been associated with various conditions, including inflammatory bowel disease, diabetes, obesity, metabolic syndrome, and cancer.^16^ Recent research has identified a significant link between gut microbiota and tumor development. Previous studies have established that microbial dysbiosis plays a critical role in the pathogenesis of gastrointestinal tumors, such as colorectal and liver cancers, as well as in tumors outside the gastrointestinal tract, including skin cancer, oral cancer, lung cancer, and gynecological tumor.^17^, ^18^ The relationship between the gut microbiota and cancer, along with its impact on the underlying mechanisms of cancer development, is highly complex and multifaceted. At present, there is no consensus on the relationship between gut microbiota and the risk of laryngeal cancer. The evidence remains inconclusive, highlighting the need for additional research to clarify this potential association and its underlying mechanisms.

Mendelian Randomization (MR) is a research methodology that leverages genetic variations, specifically Single Nucleotide Polymorphisms (SNPs) identified through Genome-Wide Association Studies (GWAS), as Instrumental Variables (IVs) to infer causal relationships between exposures and disease outcomes. This approach reduces potential biases, such as confounding and reverse causation, thereby providing more robust evidence for causality in epidemiological studies.^19^, ^20^ Therefore, to further explore the potential link between gut microbiota and the risk of laryngeal cancer, we conducted a Mendelian Randomization (MR) study. In this investigation, we utilized gut microbiota data from Genome-Wide Association Studies (GWAS) performed by the MiBioGen consortium as the exposure variables, with laryngeal cancer serving as the outcome variable. The objective was to assess the causal relationship between gut microbiota composition and the risk of developing laryngeal cancer.

## Methods

**Fig. 1:**
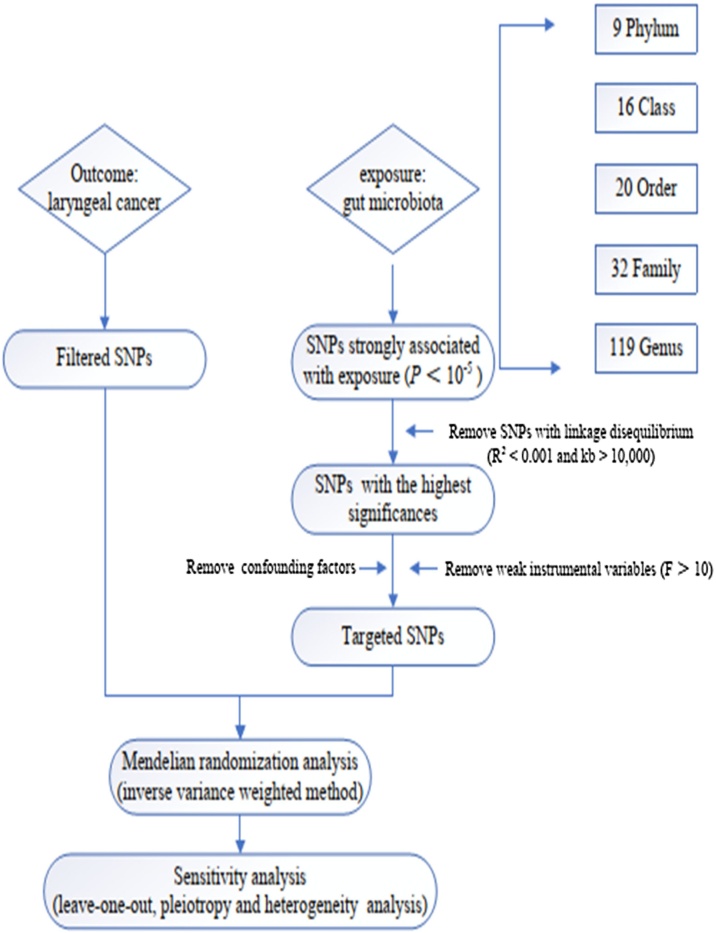
Flow chart of this study.

The flowchart of our study is shown in Fig. 1.

### Study population and data sources

Genetic summary statistics for laryngeal cancer were derived from a Genome-Wide Association Study (GWAS) including 273 cases and 372,016 controls of European ancestry, with the original data sourced from the UK Biobank, a large-scale prospective study involving over half a million participants from the United Kingdom, the UK Biobank dataset provides extensive data on phenotypic characteristics, genetic profiles, and genome-wide genotyping. This comprehensive dataset has been made publicly accessible for research purposes.^21^, ^22^ Genetic summary data for gut microbiota were acquired from the MiBioGen consortium. The data sources in this study are detailed in Table 1. The data in this study consist of publicly available GWAS summary statistics that have already received ethical approval, thereby obviating the requirement for a separate ethics application from our side.

**Table 1:** Details of the GWAS and datasets used in the study.

Exposure/outcome	Id	Sample size	Population	Portal website
Human gut microbiome		18,340 participants	Mixed	https://mibiogen.gcc.rug.nl
Laryngeal cancer	ieu-b-4913	273 cases and 372,016 controls	European	https://gwas.mrcieu.ac.uk/

### Selection of instrumental variables

Gut microbiota was selected as our exposures. Initially, we excluded 15 bacterial traits that lacked specific names, resulting in a total of 196 bacterial traits retained for analysis. These include 9 Phylum, 16 Class, 20 Order, 32 Family and 119 Genus. SNPs significantly associated with gut microbiota were chosen as IVs in this analysis. To ensure a sufficient number of SNPs as IVs with the desired level of significance, we employed a genome-wide significance threshold of *p* < 5 × 10^−5^ for selecting exposure variables. To exclude SNPs exhibiting linkage disequilibrium (LD), we applied a threshold of r^2^ < 0.001 and kb > 10,000. Additionally, palindromic SNPs, SNPs with inconsistent alleles between exposure and outcome samples (i.e., A/G vs. A/C), or SNPs with a *p-*value less than 0.05 for the outcome were removed to ensure consistent alignment of allele effects between the exposure and outcome variables.^23^ To further assess the validity of the selected IVs, we calculated the *R^2^* and F-statistic for each SNP using the following formulae: R2=2×β2 × EAF×(1-EAF); F = (N-K-1) × R2/(1-R2) × k, where “EAF” refers to the minor allele frequency of the SNPs utilized as IVs, “*R*²” represents the proportion of the variance in the exposure explained by the IVs, “N” denotes the sample size, and “K” indicates the number of IVs employed in the analysis. SNPs with an F-statistic greater than10 were retained for subsequent analysis, indicating that the selected SNPs had a robust predictive capability for the exposure variable, thereby minimizing the influence of weak instruments in the analysis.^24^ Subsequently, we systematically examined the selected SNPs for potential confounding factors associated with laryngeal cancer, including smoking, alcohol consumption, and HPV infection using the GWAS Catalog (https://www.https://www.ebi.ac.uk/gwas). If the selected SNPs are associated with smoking, alcohol consumption, or HPV infection (at a significance threshold of *p* < 10^−^⁵), those SNPs will be excluded, the remaining SNPs will then undergo subsequent analysis.

### Mendelian randomization analysis

The analysis is based on three key assumptions: (1) The instruments are strongly correlated with the exposure of interest; (2) The instruments are independent of the outcome, except through their relationship with the exposure, and are not influenced by any confounding factors; and (3) The instruments influence the outcome solely through their effect on the exposure, without any direct impact on the outcome. To investigate the causal relationship between gut microbiota and laryngeal cancer, five regression models were used: Inverse Variance Weighted (IVW) method, Weighted Median Estimation (WME), MR-Egger regression, simple mode method, and weighted mode method. The IVW method was employed as the primary analytical approach, while the remaining four models were used as supplementary methods to support and validate the findings. The IVW method enables the estimation of the predictive value of genetic factors that influence the relationship between the exposure and outcome variables, as reflected by the effect size (β). This approach calculates the causal effect by aggregating estimates from multiple genetic instruments, with each estimate derived using the Wald ratio for individual instruments, thereby providing a robust overall estimate of the causal relationship. Subsequently, we assessed the results of our mendelian randomization study. First, we evaluated the sensitivity of the remaining SNPs by sequentially removing individual SNPs, applying the leave-one-out method. Next, heterogeneity in the IVW estimates was assessed using Cochran's *Q* test and the MR-PRESSO test. Finally, pleiotropy in the IVs was evaluated through the MR Egger regression.

### Statistical analysis

All mendelian randomization analysis were conducted using the “TwoSampleMR” and “MR-PRESSO” packages in *R* (version 4.30). A threshold of *p* < 0.05 was applied to indicate statistical significance in the evaluation of the results.

## Results

### The results of instrumental variables selection

We excluded 15 bacterial traits that lacked specific names, yielding a final dataset of 196 bacterial traits. Applying a screening criterion for IVs with a significance threshold of *p* < 1.0 × 10^−5^ and r^2^ < 0.001 and kb > 10,000; we identified a total of 2,601 SNPs from the 196 intestinal microbiota. This included 124 SNPs from 9 phyla, 223 SNPs across 16 classes, 279 SNPs within 20 orders, 444 SNPs corresponding to 32 families, and 1,531 SNPs linked to 119 genera. The F-statistics for all SNPs exceeded the threshold of 10, indicating that the IVs employed in this study are robust and not prone to weak instrument bias.

### Causal effects of gut microbiota on laryngeal cancer

**Fig. 2:**
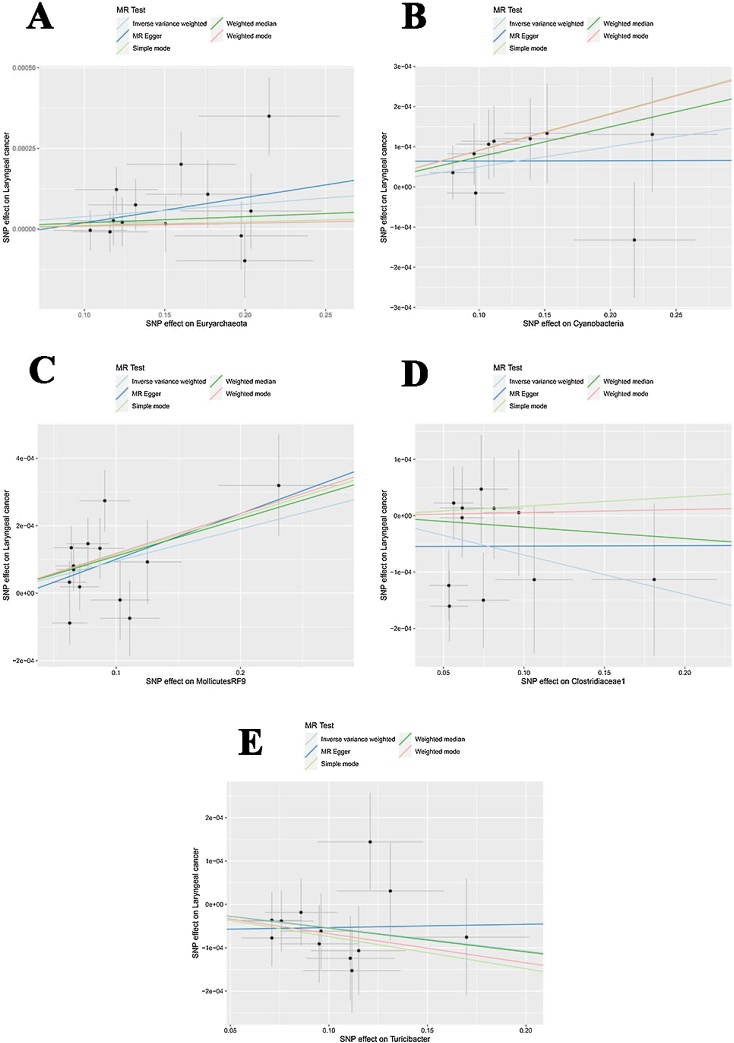
Scatter plots for causal SNPs effect of gut microbiota on laryngeal cancer. Each black point representing each SNP on the exposure (horizontal-axis) and on the outcome (vertical-axis) is plotted with error bars corresponding to each Standard Error (SE). (A) Euryarchaeota; (B) Cyanobacteria; (C) MollicutesRF9; (D) Clostridiaceae1; (E) Clostridiaceae1.

Mendelian randomization analysis was conducted on 196 bacterial taxa using the IVW method, with SNPs filtered at a significance level of *p* < 0.05. The analysis identified 58 SNPs across 5 bacterial taxa that exhibit a causal relationship with laryngeal cancer, and none of these SNPs were associated with smoking, alcohol consumption, or HPV infection (Table 2). These taxa encompass 2 phylum (22 SNPs), 1 order (13 SNPs), 1 family (11 SNPs), and 1 genus (12 SNPs), specifically including Euryarchaeota, Cyanobacteria, MollicutesRF9, Clostridiaceae1, and Turicibacter. The MR results were shown in Fig. 2A‒E. The results of IVW method indicated that increased abundances of Mollicutes RF9 (OR = 1.0010, 95% CI 1.0003–1.0016, *p* = 0.0027), Euryarchaeota (OR = 1.0004, 95% CI 1.0001–1.0007, *p* = 0.0234) and Cyanobacteria (OR = 1.0005, 95% CI 1.0000‒1.0009, *p* = 0.0464) was associated with an increased risk of laryngeal cancer, moreover, higher genetically predicted levels of Clostridiaceae1 (OR = 0.9993, 95% CI 0.9986–0.9999, *p* = 0.0463) and Turicibacter (OR = 0.9995, 95% CI 0.9989–0.9999, *p* = 0.0384) were associated with a reduced risk of laryngeal cancer (Table 2, Fig. 2A‒E). Among these associations, the relationship between Mollicutes RF9, Euryarchaeota, and laryngeal cancer risk demonstrated the strongest statistical significance (*p* = 0.0027; *p* = 0.0234), suggesting a potentially more robust effect compared to other taxa. Meanwhile, the observed associations for Cyanobacteria (*p* = 0.0464) and Clostridiaceae1 (*p* = 0.0463) were borderline significant, indicating that these findings should be interpreted with caution and validated in larger cohorts

**Table 2:** The associations between genetically determined 5 bacterial traits with the risk of laryngeal cancer through IVW method.

Gut microbiota	Level	Number of SNPs	OR (95% CI)	p
Euryarchaeota	Phylum	13	1.0004 (1.0001–1.0007)	0.0234
Cyanobacteria	Phylum	9	1.0005 (1.0000–1.0009)	0.0464
MollicutesRF9	Order	13	1.0010 (1.0003–1.0016)	0.0027
Clostridiaceae1	Family	11	0.9993 (0.9986–0.9999)	0.0463
Turicibacter	Genus	12	0.9995 (0.9989–0.9999)	0.0384

CI, Confidence Interval; OR, Odds Ratio; SNP, Single Nucleotide Polymorphism.

### Sensitivity analysis

**Fig. 3:**
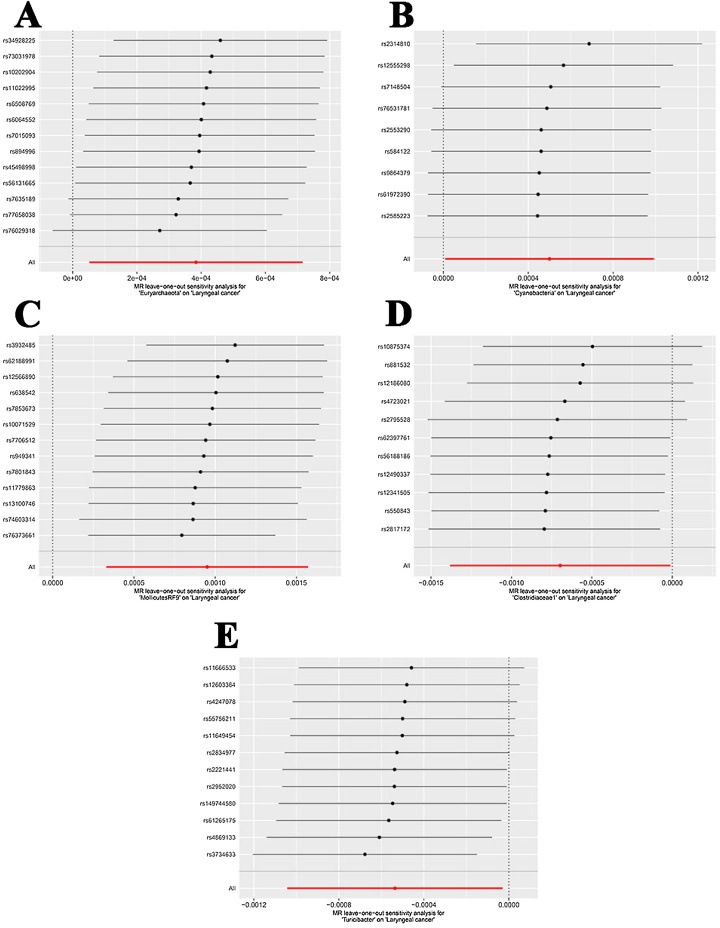
Forest plots of Leave-one-out analyses for causal SNP effect of gut microbiota on laryngeal cancer. The error bars indicate the 95% Confidence Interval (95% CI). (A) Euryarchaeota; (B) Cyanobacteria; (C) MollicutesRF9; (D) Clostridiaceae1; (E) Clostridiaceae1.

The results of Cochran's *Q* test and MR-PRESSO indicated no significant heterogeneity among the selected IVs (Table 3), suggesting that the IVs are consistent across the analyzed models. Furthermore, the MR Egger regression analysis demonstrated no evidence of horizontal pleiotropy within the examined bacterial taxa (Table 3), indicating that the causal estimates are unlikely to be confounded by pleiotropic effects. Additionally, the leave-one-out sensitivity analysis showed no notable outliers among the selected IVs (Fig. 3A‒E), reinforcing the robustness and reliability of the MR analysis results.

**Table 3:** Sensitivity analysis of the gut microbiota on laryngeal cancer.

Gut microbiota	Level	Heterogeneity test			Horizontal pleiotropy test (*p*)
		IVW Q_*p-*value	MR Egger Q_*p-*value	MR-PRESSO_Global (p)	
Euryarchaeota	Phylum	0.3691	0.3171	0.4020	0.5898
Cyanobacteria	Phylum	0.7695	0.7302	0.7690	0.5166
MollicutesRF9	Order	0.1524	0.1192	0.2100	0.6685
Clostridiaceae1	Family	0.3568	0.3170	0.4190	0.4968
Turicibacter	Genus	0.8474	0.8142	0.8690	0.5621

IVW, Inverse Variance Weighted.

## Discussion

To date, no published studies have investigated the genetic causal relationship between gut microbiota and the risk of laryngeal cancer, and there remains a lack of consensus on the association between gut microbiota and laryngeal cancer risk. Our study fills this gap and provides novel insights into the potential role of gut microbiota in laryngeal cancer susceptibility through MR analysis utilizing GWAS data. Our findings revealed a positive association between the presence of Euryarchaeota (OR = 1.0004, 95% CI 1.0001–1.0007, *p* = 0.0234), Cyanobacteria (OR = 1.0005, 95% CI 1.0000‒1.0009, *p* = 0.0464), and Mollicutes RF9 (OR = 1.0010, 95% CI 1.0003–1.0016, *p* = 0.0027) and an increased risk of laryngeal cancer. Conversely, Clostridiaceae1 (OR = 0.9993, 95% CI 0.9986–0.9999, *p* = 0.0463) and Turicibacter (OR = 0.9995, 95% CI 0.9989–0.9999, *p* = 0.0384). While our findings suggest potential causal effects, it is essential to distinguish between true biological effects and statistical correlations. The Odds Ratios (ORs) observed were modest, implying that gut microbiota may act as a contributing factor rather than a primary driver of laryngeal cancer.

Euryarchaeota is a major phylum within the domain Archaea, comprising a diverse array of microorganisms such as methanogens, extreme halophiles, thermophiles, and certain acidophiles. This phylum plays a pivotal role in the gut microbiome, contributing to critical metabolic functions that are essential for host health. Members of Euryarchaeota are integral to the gut's microbial ecosystem, engaging in processes such as methanogenesis and nutrient cycling, thereby influencing the overall balance and function of the microbiome and supporting the host’s physiological well-being.^25^, ^26^ Although Euryarchaeota are less commonly studied in the context of human health compared to bacteria, recent research suggests potential roles in cancer and tumor promotion: Microbial dysbiosis, characterized by an imbalance in the microbial community, can significantly disrupt interactions with other gut bacteria, thereby affecting host metabolism, inflammatory responses, and immune function. Such disruptions may contribute to the creation of a tumor-promoting microenvironment.^27^, ^28^ Additionally, methanogenic archaea have been implicated in the development of low-grade chronic inflammation, a recognized risk factor for cancer. Chronic inflammation within the gut can promote cellular proliferation and genomic instability, thereby elevating the risk of colorectal and other cancers.^29^, ^30^ Cyanobacteria, commonly known as blue-green algae, have recently been identified as part of the human gut microbiome. These microorganisms are capable of producing toxins such as microcystins and nodularins, which are known for their hepatotoxic effects and potential to induce carcinogenesis.^31^ Cyanobacteria can also generate Reactive Oxygen Species (ROS), leading to oxidative stress ‒ a well-established mechanism that contributes to DNA damage, genetic mutations, and the activation of oncogenic pathways.^32^ Furthermore, the presence of Cyanobacteria in the gut microbiome may disrupt the balance between beneficial and harmful bacteria, a condition associated with an increased risk of cancer.^33^ Mollicutes RF9 is a subgroup within the class Mollicutes, which are characterized by their lack of a cell wall and their parasitic or commensal lifestyle in various hosts, including humans. Mollicutes RF9 is primarily involved in inducing chronic inflammation, disrupting the balance of the gut microbiome, and modulating host immune responses in ways that facilitate tumor immune evasion.^34^, ^35^, ^36^ Additionally, it interferes with metabolic processes and produces toxins that can impair normal cellular functions, promote DNA damage, and alter critical cell signaling pathways.^37^ These actions collectively position Mollicutes RF9 as a significant risk factor for tumor development. Our study findings suggest that Euryarchaeota, Cyanobacteria, and Mollicutes RF9 may elevate the risk of laryngeal cancer, indicating a potentially detrimental role in laryngeal cancer development. This association may be mediated through the mechanisms previously described, including chronic inflammation, disruption of microbial balance, immune modulation, and metabolic interference.

Prior to this study, there have been no reported investigations examining the association between Clostridiaceae1, Turicibacter, and laryngeal cancer. Our study found that Clostridiaceae1 and Turicibacter were negatively associated with the risk of laryngeal cancer, suggesting a potential protective role of these gut microbiota against the development of laryngeal cancer. Clostridiaceae1 is a family within gut microbiota, and Turicibacter is a genus of bacteria. They are known for their beneficial roles in cancer suppression, including the production of Short-Chain Fatty Acids (SCFAs), modulation of the immune system, and maintaining gut barrier integrity.^38^, ^39^, ^40^, ^41^, ^42^, ^43^ It is evident that Clostridiaceae1 and Turicibacter may exert a protective effect against the development of laryngeal cancer, potentially through the modulation of specific pathways. Euryarchaeota and Mollicutes RF9 have been linked to chronic inflammation through their ability to induce the production of pro-inflammatory cytokines,^44^ such as IL-6 and TNF-α. Cyanobacteria can produce toxic metabolites, including microcystins and nodularins, which may disrupt normal cellular function and promote tumorigenesis. Mollicutes RF9 has been associated with host metabolic processes by altering lipid metabolism and generating Reactive Oxygen Species (ROS), leading to oxidative stress and DNA damage, and immunosuppressive effects that allow malignant cells to escape immune surveillance.^44^ Clostridiaceae1 and Turicibacter are known for their role in producing anti-inflammatory cytokines, such as IL-10, which help suppress excessive inflammation and maintain immune homeostasis. Moreover, Clostridiaceae1 is involved in the production of Short-Chain Fatty Acids (SCFAs), particularly butyrate, which has been shown to exert anti-tumor effects by promoting cell differentiation, inducing apoptosis in cancer cells, and inhibiting Histone Deacetylases (HDACs), and Turicibacter has been implicated in the regulation of T-cell responses, particularly in enhancing anti-tumor immunity.

Our study identified five gut microbiota that may be implicated in the development of laryngeal cancer; Although the causal relationship is relatively weak, the results remain of reference value. Our findings open avenues for microbiota-based interventions in laryngeal cancer prevention and treatment: Future studies could explore the potential of probiotics, dietary modifications, and microbiome-targeted therapies to modulate gut microbiota and reduce cancer risk, and specific bacterial signatures may serve as non-invasive biomarkers for laryngeal cancer screening, aiding in early diagnosis and improved prognosis, and the gut microbiome has been implicated in modulating responses to chemotherapy and immunotherapy. Additionally, a deeper understanding of these underlying pathways will enhance our knowledge of laryngeal cancer pathogenesis and facilitate the development of more precise strategies for its prevention, early detection, and treatment.

This study possesses several strengths: First, we established causal relationships a large, high-quality GWAS database, thereby strengthening the credibility of the identified associations. Second, our Mendelian Randomization analysis identified key candidate microbial taxa that warrant further functional investigation, offering potential insights into novel therapeutic and preventive strategies targeting specific gut microbiota for laryngeal cancer. However, our study has several limitations. First, the research primarily relied on GWAS summary data from European populations, with only a limited representation of gut microbiota data from other ethnic groups. Since microbiota composition varies significantly across different populations, this could affect the generalizability of our findings. Moreover, GWAS-based microbiome studies often face challenges in defining causal microbial signatures due to the complex nature of host-microbiota interactions. The relatively small sample size (273 cases of laryngeal cancer) may limit the statistical power of our study. Given the complexity of cancer development and the variability in microbiota composition among individuals, larger and more diverse datasets will be necessary in future studies to validate our findings and strengthen causal inferences. Second, the development of laryngeal cancer is a multifactorial process influenced by complex interactions between genetic and environmental factors, including the gut microbiota composition. While MR analysis helps mitigate some confounding, it does not completely eliminate the influence of gene-environment and gene-diet interactions. For example, dietary habits, smoking, alcohol consumption, and HPV infection can significantly shape gut microbiota composition, potentially influencing the associations observed in our study. Moreover, the reliability of genetic instruments used in MR analysis depends on the strength and specificity of the genetic variants associated with gut microbiota. Many microbial traits identified in GWAS are based on relatively small sample sizes, which may lead to weak instrument bias, thereby reducing the power of causal inference. Additionally, potential pleiotropic effects, where genetic variants influence multiple traits independently of microbiota composition, could introduce additional biases in MR estimates. To minimize these concerns, future research should utilize larger GWAS datasets with well-characterized genetic instruments to strengthen causal inference. As such, we cannot rule out the potential confounding effects of gene-diet and gene-environment interactions on our results. To further validate the functional roles of the identified microbiota, we plan to conduct in vitro experiments to explore the impact of modulating these microbiotas on laryngeal cancer, focusing on changes in cell proliferation, migration, and apoptosis. Additionally, we intend to perform in vivo studies using animal models to assess the therapeutic potential of targeting these microbiotas in the context of laryngeal cancer.

## Conclusion

In summary, this study provides a comprehensive analysis of the potential causal relationship between gut microbiota and laryngeal cancer. We identified five specific gut microbiota that are associated with the risk of developing laryngeal cancer. These findings offer novel insights and open new avenues for future strategies in the prevention and treatment of laryngeal cancer.

## CRediT authorship contribution statement

Conceptualization: Kaiyan Yi and Yu Huang. Data curation: Yu Huang. Project administration: Lingling Zhou. Writing-original draft: Lingling Zhou. Writing-review and editing: Yun Jiang.

## Consent for publication

Not applicable.

## Consent to participate

Not applicable.

## Ethics approval

Not applicable.

## Funding

This study received funding support from the Key Disciplines of Huadong Hospital (ZDXK2214).

## Data availability statement

In this study, publicly available datasets were utilized for the analysis. The original data are accessible at the following URL: https://gwas.mrcieu.ac.uk/datasets/. For additional information or specific inquiries, please contact the corresponding author.

## Declaration of competing interest

The authors declare no conflicts of interest.
